# Characterization of human mitochondrial ferritin promoter: identification of transcription factors and evidences of epigenetic control

**DOI:** 10.1038/srep33432

**Published:** 2016-09-14

**Authors:** Michela Guaraldo, Paolo Santambrogio, Elisabetta Rovelli, Augusta Di Savino, Giuseppe Saglio, Davide Cittaro, Antonella Roetto, Sonia Levi

**Affiliations:** 1 San Raffaele Scientific Institute, Division of Neuroscience, 20132 Milano, Italy; 2 University of Torino, Department of Clinical and Biological Sciences, AOU San Luigi Gonzaga, 10043 Orbassano, Torino, Italy; 3 San Raffaele Scientific Institute, Center for Translational Genomics and Bioinformatics, 20132 Milano, Italy; 4 University Vita-Salute San Raffaele, 20132 Milano, Italy

## Abstract

Mitochondrial ferritin (FtMt) is an iron storage protein belonging to the ferritin family but, unlike the cytosolic ferritin, it has an iron-unrelated restricted tissue expression. FtMt appears to be preferentially expressed in cell types characterized by high metabolic activity and oxygen consumption, suggesting a role in protecting mitochondria from iron-dependent oxidative damage. The human gene (*FTMT*) is intronless and its promoter region has not been described yet. To analyze the regulatory mechanisms controlling *FTMT* expression, we characterized the 5′ flanking region upstream the transcriptional starting site of *FTMT* by *in silico* enquiry of sequences conservation, DNA deletion analysis, and ChIP assay. The data revealed a minimal promoter region and identified the presence of SP1, CREB and YY1 as positive regulators, and GATA2, FoxA1 and C/EBPβ as inhibitors of the transcriptional regulation. Furthermore, the *FTMT* transcription is increased by acetylating and de-methylating agent treatments in K562 and HeLa cells. These treatments up-regulate FtMt expression even in fibroblasts derived from a Friedreich ataxia patient, where it might exert a beneficial effect against mitochondrial oxidative damage. The expression of *FTMT* appears regulated by a complex mechanism involving epigenetic events and interplay between transcription factors.

Mitochondria are the sites in which iron is transformed into heme and Fe-S clusters (ISC) by specific biosynthetic pathways^1^,^2^. For this reason these organelles are the major users of cellular iron and, similarly to the cell, relies on iron transport, storage, and regulatory proteins to maintain iron homeostasis^3^. One of them, mitochondrial ferritin (FtMt) belongs to the family of ferritins, the iron storage proteins, and exerts its role specifically in mitochondria, where it is efficiently imported and localized inside the matrix^4^.

Structure and function of FtMt are similar to the cytosolic ferritin with some peculiarity^5^. Its 3D structure results analogous to that of human H-ferritin (FtH). Besides, its biochemical properties are remarkably similar to those of FtH, except for its ferroxidase activity^6^. In fact, iron binding, oxygen consumption and proton production kinetic experiments comparing FtMt and FtH revealed striking differences between the two proteins in iron oxidation and hydrolysis chemistry, despite their similar ferroxidase centers^7^. However, FtMt acts as an efficient ferritin by readily incorporating and oxidizing iron *in vitro*^7^.

Previous functional studies on cellular models indicated that FtMt bound mitochondrial iron and its expression had a profound effect on cellular iron homeostasis, since it induced iron delocalization from cytosol to mitochondria^8^,^9^,^10^,^11^,^12^. The *in vivo* data on *Ftmt−/−* mouse models revealed only minor defects: i) the sideroblast/siderocyte formation in mice fed vitamin B6 (pyridoxine) deprivation diet^13^ and ii) the higher sensitivity of heart mitochondria to the toxicity of doxorubicin^14^.

Mitochondrial ferritin is encoded by a nuclear gene (*FTMT*) on chromosome 5q23.1. Genes homologous to *FTMT* have been identified in plants^15^,^16^, in insect, as *Drosophila melanogaster*^17^ and in mammals, like chimpanzee, mouse, rat and dog^18^. *FTMT* is an intronless gene, lacking the typical TATA or CCAAT box upstream the ATG start codon and codifies for a precursor peptide with a mitochondrial targeting signal^4^,^18^. This DNA region belongs to a group of non-X-linked bona fide promoter CpG island that is densely methylated in normal somatic tissues^19^. In particular, the 220 base pairs long segment upstream the ATG codon is characterized by a highly methylated GC-rich content^19^.

Despite of the relative large amount of data on FtMt functional role, little evidences have been collected on the regulation of its expression as well as on the mechanisms of its cell/tissue specific expression. In contrast with the cytosolic ferritin, *FTMT* mRNA does not contain any functional IRE sequence^18^, meaning that its expression is not iron dependent. In mammalians, it shows a tight tissue-specific expression pattern^18^. In mouse, *FTMT* expression is restricted to a limited number of cell types with a pattern apparently linked to the oxidative metabolic activity of the cells, suggesting that it might protect the mitochondria from iron-dependent oxidative damage rather than be associated to iron storage function^20^. In Sideroblastic Anemia patients, *FTMT* is highly expressed in ring sideroblasts where it detoxifies mitochondrial iron overload caused by defective heme synthesis^21^. Enhanced *FTMT* expression is demonstrated in brains of Alzheimer’s Disease (AD)^22^ and in Restless Legs Syndrome (RLS) affected patients^23^. In the case of AD, it was proposed that overexpression of *FTMT*, induced by oxidative stress, decreased the amount of toxic AβPP peptide^24^, while in RLS its enhancement could be detrimental and contribute to the pathologic cytosolic iron deprivation^23^. Furthermore, the increased expression of *FTMT* was detected in cardiomyocytes of Friedreich Ataxia (FRDA) patients^25^. A more recent study showed a downregulation of *FTMT* in Neuroblastoma and in Neurospongioma, where it has been proposed that *FTMT* could be used as a target to inhibit neuronal cell proliferation through its overexpression^26^. However, *FTMT* expression may also be detrimental, as showed in K562 erythroid cells where its overexpression reduced JAK/STAT signaling and increased apoptosis^27^,^28^.

In this work, we investigate the transcriptional regulation of *FTMT* and we identify the putative promoter region, comprising the minimal promoter as well as a positive and a negative transcriptional factors target regions. We also explored the possibility of epigenetic control as responsible for *FTMT* silencing in a tissue-specific manner. Furthermore, given the protective role of FtMt described in FRDA^10^,^11^, we analyzed a hypothetic epigenetic intervention to increase *FTMT* expression in FRDA fibroblasts.

## Results

### Identification of putative promoter region of *FTMT*

**Figure 1:**
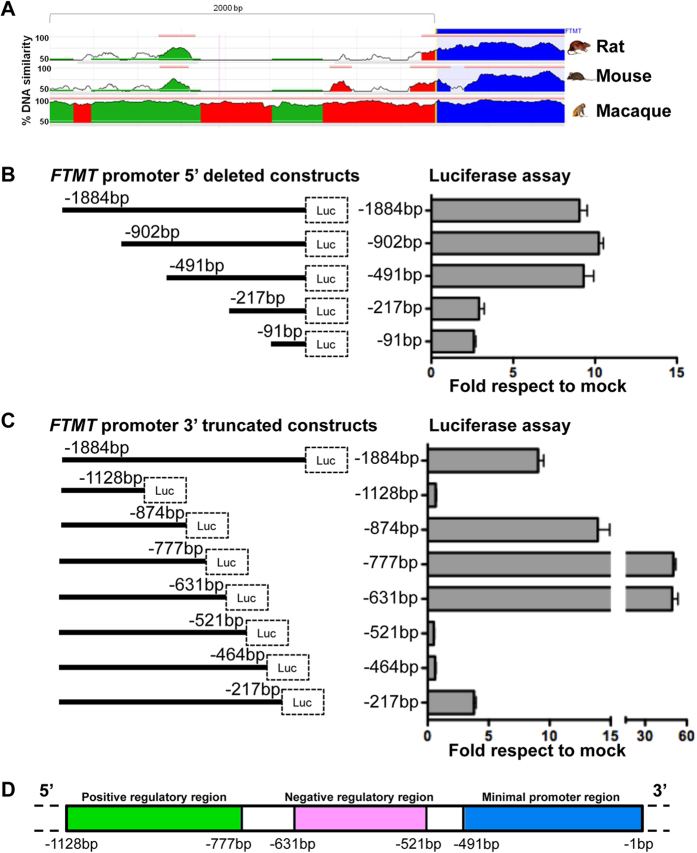
Promoter identification by in silico analysis and luciferase assay. (**A**) Mammalian conservation of *FTMT* 5′ genomic sequence, corresponding to chr5:121185610:121189119 on Human GRCh37 Assembly (hg19). The conservation summary is adapted from ECR Browser^47^. (**B**) Luciferase assay on promoter sequence with 5′ and **(C)** 3′ deletions to identify the promoter activity. Left panels show the scheme of the constructs. Right panels show the quantification of luciferase emission with the indicated constructs normalized to empty vector. The plots represent the mean +/−SD of three independent experiments. (**D**) Scheme showing the position of positive (green) and negative (pink) regulatory and minimal promoter (blue) regions, resulting from luciferase assay.

To identify the putative promoter region of *FTMT* gene we looked for conserved consensus sequences upstream the transcription-starting site among different species by *in silico* analysis. In particular, the region from −2040 base pairs to +600, corresponding to UCSC chr5:121185610:121189119 on Human GRCh37 Assembly (hg19), showed a sequence identity of 70% to mouse and 93% to macaque (Fig. 1A). We cloned the sequence −1884 bp to −1 from the transcription-starting site in front of a luciferase reporter construct and we made a series of 5′ and 3′ deletions to test their effect on luciferase expression in order to determine the region responsible for basal promoter activity. These vectors were transfected into HeLa cells and we analyzed their promoter activity compared to the cells transfected with the empty vector (mock) (Fig. 1B,C). In the 5′ deletions experiments the −1884 construct had an activity of 10 fold respect to mock, which we used as value reference (Fig. 1B). The segment −902/−1 and fragment −491/−1 showed a similar promoter activity to −1884/−1 fragment; on the contrary the −217/−1 and −91/−1 resulted in a diminished promoter activity (Fig. 1B). Analysis of 3′ truncated constructs presented complementary results. In fact, the −1884/−1128 fragment showed a strong decreased activity, while −1884/−874 had an increased activity. Finally −1884/−464 and −1884/−217 showed a weakened promoter activity (Fig. 1C). To better characterize the results obtained, we made smaller 3′ truncation, between −1128 and −464. The −1884/−777 and −1884/−631 segments showed 50 fold increased activity respect to mock, indicating the presence of a putative activator region. The −1884/−521 and −1884/−464 segments showed a strong reduction of activity indicating the presence of a putative inhibitory region (Fig. 1C). In conclusion we identified the presence of two regions: one from −1128 to −631, which contains activating regulatory elements, and one from −631 to −521 that contains suppressing regulatory elements. We also found that the 491bp fragment upstream transcription starting site contains a minimal promoter, confirmed by the absence of activity with the deletion of this region from the entire construct (−1128/−521 and −1128/−464) (Fig. 1D).

### Analysis of transcription factors for *FTMT* promoter

**Figure 2:**
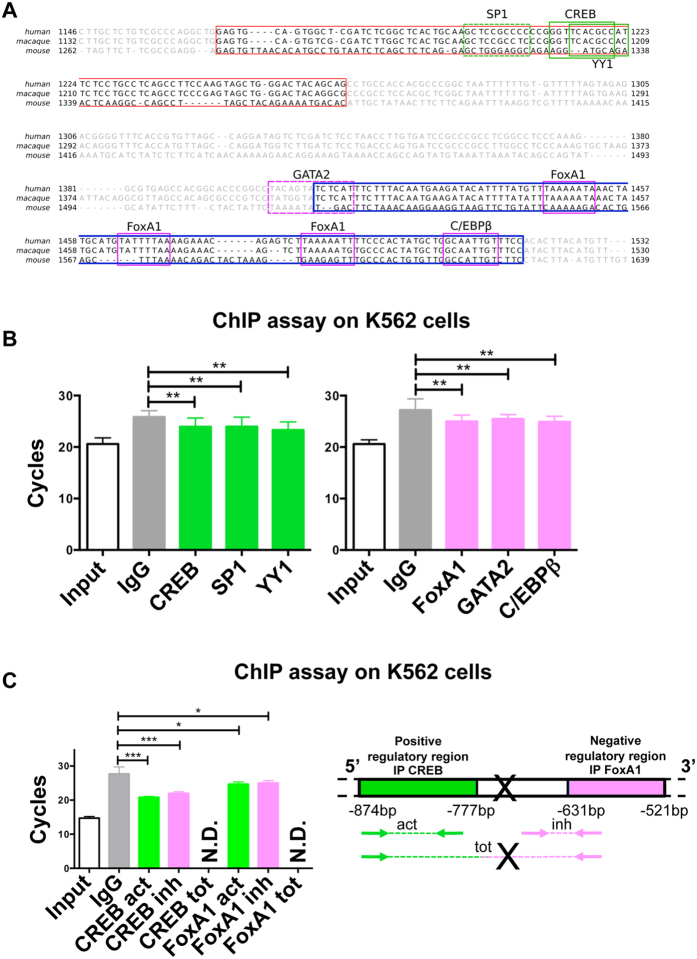
Identification of transcription factors and Chromatin Immuno Precipitation (ChIP) analysis. (**A**) Conservation of identified activator and repressor sequence in human, macaque, and mouse. In red the region containing positive regulatory elements, from −874 to −777, in blue the region comprising negative regulatory elements, from −631 to −521. The location of putative binding sites for transcription factors are boxed: positive transcription factors are in green while negative are in pink. Solid line represent transcription factors bind to positive strand DNA, while dotted line indicate the ones bind to negative strand DNA. The sequence alignment was created with ClustalW2; transcription factors were identified by MatInspector and PROMO Alggen. (**B**) Chromatin immunoprecipitation (ChIP) prepared from K562 cells with the indicated set of antibodies specific for transcription factors selected by in silico analysis of the *FTMT* promoter. The amount of co-precipitated DNA is evaluated by qRT-PCR using oligonucleotides specific for positive and negative regulatory elements. On the left panel, ChIP of activator region: CREB, SP1 and YY1 transcription factors interacting with positive regulatory elements. On the right panel, ChIP of repressor region: FOXA1, GATA2 and C/EBPβ transcription factors interacting with negative regulatory elements. IgG represents the negative control with non-specific antibodies. Input represents the positive control of chromatin without immunoprecipitation. The plots represent the mean +/−SD of four independent experiments in triplicate, analyzed with One-way Anova with Bonferroni post test, **p < 0.01. (**C**) Chromatin immunoprecipitation (ChIP) as in B, the chromatin immunoprecipitated with the indicated transcription factors interacting with positive or negative regulatory elements was amplified also with the oligonucleotides specific for the opposite and the total regulatory elements (act = activator, inh = inhibitor, tot = total regulatory elements). N.D. = not detectable. X = possible broken region. The plots represent the mean +/−SD of two independent experiments in triplicate, analyzed with One-way Anova with Bonferroni post test, *p < 0.05, ***p < 0.001.

To identify transcription factors involved in positive and negative regulation for *FTMT* gene, we made an *in silico* analysis of the sequence using ClustalW2, MatInspector and PROMO databases. We predicted transcription factors to bind to the region identified by the luciferase assay, between −1128 and −521 bp and we selected the transcription factors conserved also in mouse and macaque (Fig. 2A). Among them, in the region containing activating regulatory elements, we selected CREB and YY1, that bind to the positive strand, and SP1 that binds to the negative strand (Matrix similarity in MatInspector: CREB = 0.92, SP1 = 0.97, YY1 = 0.84 and dissimilarity margin in PROMO: CREB = not found, SP1 = not found, YY1 = 0.49%). In the region encompassing suppressing regulatory elements, we selected FoxA1 and C/EBPβ, which bind to the positive strand, and GATA2 that binds to negative strand (Matrix similarity in MatInspector: FoxA1 = 0.96, C/EBPβ = 0.92, GATA2 = not found and dissimilarity margin in PROMO: FoxA1 = 3.23%, C/EBPβ = 2.24%, GATA2 = 6.67%).

[Table t1]To confirm the results obtained by *in silico* analyses, we studied *in vitro* whether the transcription factors interact with the two regulatory regions of the *FTMT* promoter by performing chromatin immunoprecipitation (ChIP) analysis. Chromatin solutions isolated from K562 were incubated with specific antibodies for the selected transcription factors (see Materials and Methods). DNA isolated by ChIP from the K562 samples was analyzed by quantitative real time PCR (Fig. 2B), using the oligonucleotides specific for the two identified regulatory regions (activator and inhibitor, Table 2). The results showed that the regions identified in the *FTMT* promoter were occupied by the transcription factors. Indeed, the region in the *FTMT* promoter containing the activating regulatory elements was immunoprecipitated by the transcription factors CREB, SP1, and YY1 with significant difference compared to the non-specific IgG used as negative control (Fig. 2B left panel). Similarly, the region containing the suppressing regulatory elements was immunoprecipitated by the transcription factors FoxA1, Gata2 and C/EBPβ (Fig. 2B, right panel). Non-precipitated chromatin input was used as PCR positive control (Fig. 2B). In order to verify the putative interaction between the different transcription factors, we performed chromatin immunoprecipitation for two of them, one binding the activating regulatory region (CREB) and one the suppressing regulatory elements (FoxA1) (Fig. 2C, left panel). Then, we

amplified the immunoprecipitated chromatin with oligonucleotides specific for the single activating and suppressing regulatory elements (schematized in Fig. 2C, right panel) and with a pair of oligonucleotides specific for a more wide region containing both elements (schematized in Fig. 2C, right panel). The amplification with the oligonucleotides including both regulatory regions resulted undetermined, suggesting that the two regulatory regions are physically separated. On the contrary, the amplification of the suppressing regulatory elements, after immunoprecipitation with the transcriptional factor binding the activating regulatory region (and viceversa), resulted measurable, with significant difference compared to the non-specific IgG (Fig. 2C, left panel). This result implies that the transcription factors bound to activating and suppressing regulatory region interact to form transcriptional machinery complex.

### Epigenetic control of *FTMT* expression

**Figure 3:**
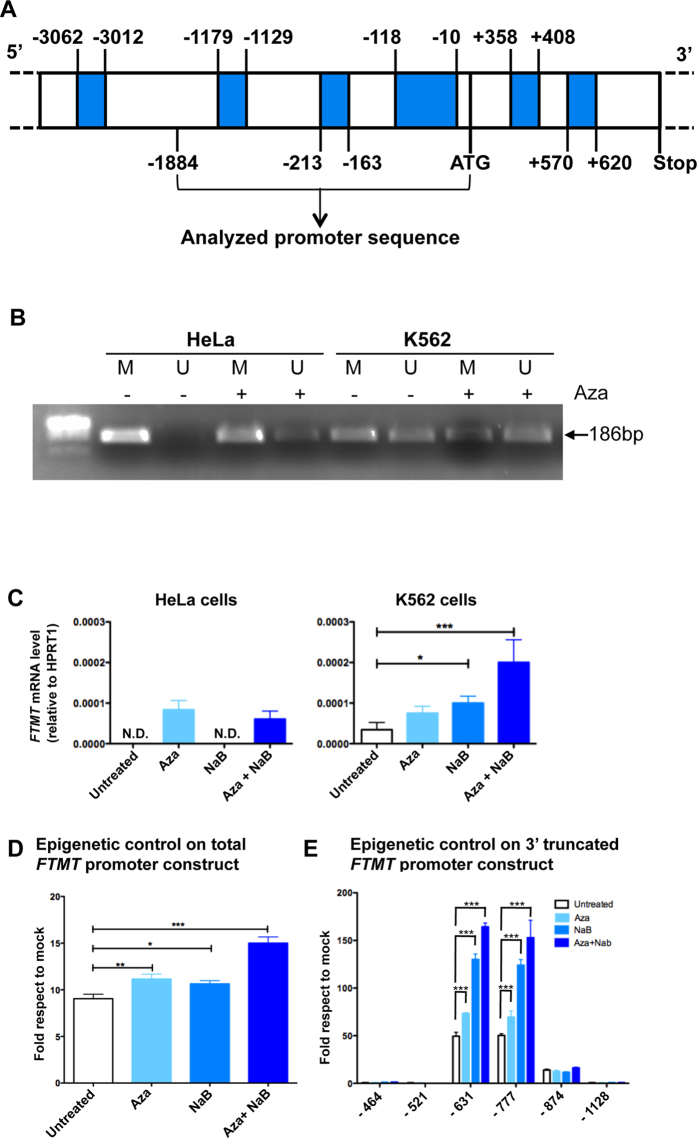
Epigenetic control of *FTMT* expression. (**A**) Schematic representation of the putative methylated regions in the *FTMT* promoter predicted by UCSC Genome Browser. Regions that can be methylated are shown in blue, numbers indicate position on the sequence respective to ATG, starting and stop codons (ATG and Stop, respectively) are indicated. (**B**) Methylation specific PCR showing methylated (M) and unmethylated (U) DNA in HeLa and K562 cells, untreated (−) or treated (+) with 5 μM Aza for 72 h. Arrow indicates PCR product size. **(C)** Expression of *FTMT* by qRT-PCR in HeLa and K562 cells untreated, treated with the demethylating agent (5 μM Aza), with the histone deacetylase inhibitor (1 mM sodium butyrate, NaB) or both for 72 h. The plots represent the mean +/−SD of three independent experiments in triplicate, analyzed with One-way Anova with Bonferroni post test, *p < 0.05, ***p < 0.001. **(D)** Luciferase assay of the putative promoter region to evaluate epigenetic contribution to *FTMT* expression in K562 cells. Luciferase activity of the full promoter region in basal condition (Untreated) or after treatment as in panel C. The plots represent the mean +/−SD of three independent experiments in triplicate, analyzed with One-way Anova with Bonferroni post test, *p < 0.05, **p < 0.01, ***p < 0.001. (**E**) Luciferase activity of promoter region with 3′ deletions upon treatment described in panel C. The plots represent the mean +/−SD of three independent experiments in triplicate, analyzed with Two-way Anova with Bonferroni post test, ***p < 0.001.

*In silico* analysis on UCSC Genome Browser reveals the presence of large CpG islands in the *FTMT* promoter that includes several CpG hotspot both in the promoter region and in the gene sequence (Fig. 3A evidenced in blue). On the basis of these evidences (Fig. 3A) and by genome-wide profiling of DNA methylation data^19^, we expected that HeLa cells should be fully methylated while K562 cells should be partially methylated. To experimentally confirm the *in silico* data, we performed methylated specific PCR and, as expected, the CpG regions in the *FTMT* promoter in HeLa cells resulted fully methylated while the CpG island in K562 was only partially methylated (Fig. 3B). Treatment of these two cell lines with 5 μM of 5-Aza-2′-deoxycytidine (Aza, an inhibitor of DNA methylation) for 72 h resulted in a decreased amount of methylated DNA both in HeLa and in K562 cell line. Consistently, mRNA of HeLa cells was not detectable in basal condition, and it appeared after Aza treatment while in K562 a detectable level of mRNA expression was found, which was up-regulated by treatment with Aza (Fig. 3C). Moreover, the supplement of 1 mM Sodium Butyrate (NaB, an histone deacetylase (HDAC) inhibitor) for 72 h had an additive effect in K562 but not in HeLa cells; in fact the combination effect of Aza and NaB in K562 cells induced a 5,8 fold increase compared to basal *FTMT* expression (Fig. 3C). For this reason we decided to use K562 cell line treated with NaB and Aza for further analysis.

To further verify if *FTMT* expression is under control of epigenetic events, we transfected the 1884 bp segment of the putative promoter region in front of a luciferase reporter sequence in K562 (Fig. 3D). Luciferase assay showed a significant increase of its expression after treatment with Aza or with NaB respect to the basal condition, further enhanced by the treatment with both substances (Fig. 3D). To better define the promoter activity we assayed the effect of the 3′ truncations constructs. Constructs with deletion of densely methylated region at 3′ (−464 and −521) did not respond to treatment with Aza and NaB. Vice versa, the simultaneous treatment with Aza and NaB in −631 bp and in −777 bp fragments induced more than 150 fold increase of luciferase activity (Fig. 3E).

### *FTMT* expression in FRDA fibroblasts

**Figure 4:**
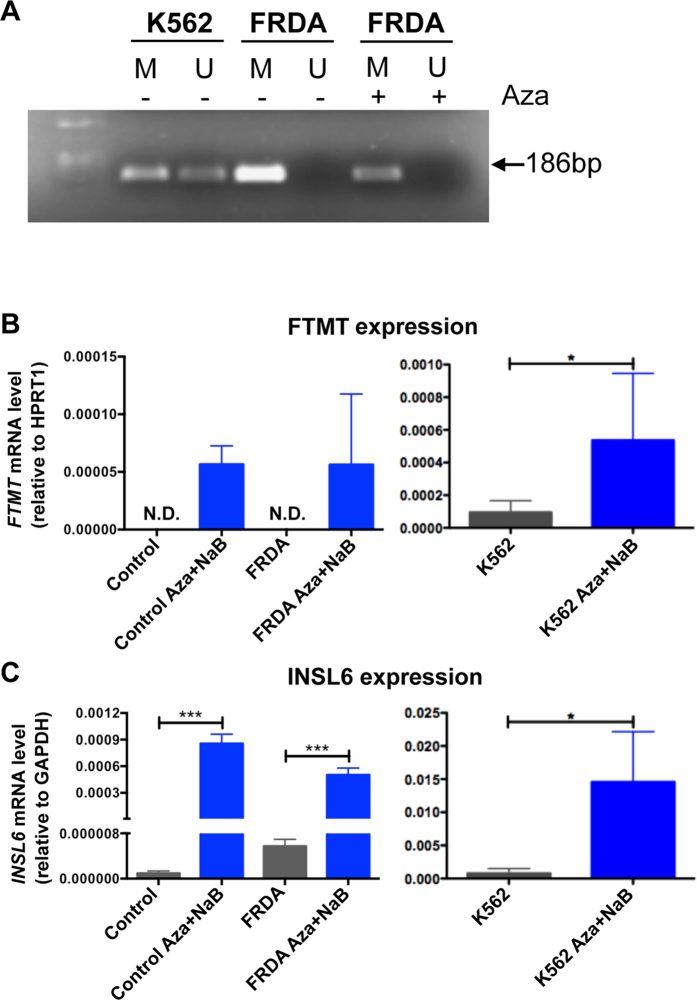
*FTMT* expression in Friedreich ataxia fibroblasts and K562 cells. (**A**) Methylation specific PCR showing methylated (M) and unmethylated (U) DNA in untreated (−) K562 cells and in primary fibroblasts from a FRDA patient untreated (−) or treated (+) with 5 μM Aza for 72 h. Arrow indicates product size. **(B)** mRNA level of *FTMT* in control and FRDA fibroblasts and K562 cells treated for 72 h with 5 μM Aza and 1 mM NaB analyzed by qRT-PCR. **(C)** The expression of *INSL6* gene is analyzed to confirm effective demethylation. The plots represent the mean +/−SD of three independent experiments in triplicate, analyzed with Mann-Whitney test *p < 0.05, ***p < 0.001.

To evaluate the epigenetic control of *FTMT* in pathological condition, we checked the epigenetic landscape in a cell line obtained from a FRDA patient compared with that of K562 cells through methylation specific PCR (Fig. 4A). *FTMT* resulted to be fully methylated at basal level in control and FRDA patient, but we can appreciate a significant decrease of the amount of methylated DNA after Aza treatment (Fig. 4A). Surprisingly, we were not able to detect a correspondent increase of demethylated DNA, may be due to the low sensitivity of the experimental approach (Fig. 4A). Nevertheless, the *FTMT* mRNA increment become evident at quantitative level in an healthy subject (control) and FRDA fibroblasts after treatment with 5 μM Aza and 1 mM NaB for 72 h (Fig. 4B). In fact, the analysis of *FTMT* expression by qRT-PCR showed that untreated control and FRDA cells had no detectable *FTMT* transcript, while demethylation and acetylation increased the expression of *FTMT*, even if at lower level than in K562 in basal condition (Fig. 4B). The control of efficacy of demethylation treatment was checked through the increment of INSL6 expression, a gene that is epigenetically regulated (Fig. 4C)^19^,^29^. Thus, the comparison between the *FTMT* expression in control and FRDA fibroblasts and K562 cells indicated that the treatment to modify the epigenetic control is less efficient in FRDA fibroblasts respect to the erythroid cells (Figs 3B and 4A,B).

## Discussion

The data previously collected on the physiological role of FtMt^5^ and its peculiar distribution pattern^20^ suggested that its expression might be regulated at transcriptional level in a specific tissue- and cell- type manner. An example of this uneven tissue distribution is its high expression in mouse testis, where the protein is mainly localized in spermatozoa and it is easily detectable by specific immunoassay, while the same assay is unable to reveal its presence in almost all of the other tissues^20^. In order to elucidate *FTMT* expression regulation we focused our attention on the 2000 bp upstream the ATG codon, where we identify the *FTMT* promoter region. By cloning the *FTMT* upstream genomic region with higher conservation between human and mouse, we determined the sequence responsible for basal promoter activity. It is contained in the 491 bp fragment upstream the transcriptional start site, as confirmed by the absence of luciferase activity in case of deletion of this region from the experimental construct. We also found two other pivotal regions: one that contains activating regulatory elements and the other one that contains inhibitory regulatory elements. Deleting the region corresponding to activator, in fact, we had no luciferase activity, meaning that proteins bind this DNA sequence to promote the transcription of *FTMT*. On the contrary, eliminating the region corresponding to inhibitor, we observed an increment of luciferase activity, suggesting that within this deleted region some factors prevent the transcription. To identify transcription factors binding these *FTMT* regulatory regions, we performed an in silico analysis and selected some of them depending on the score of matches in DNA sequences, on their conservation in mouse *FTMT* 5′ region and on their functional features (Fig. 2A). On this basis, among the identified transcription factors we selected the six of which we experimentally confirmed the binding. From the analysis of DNA region containing the activating regulatory elements, we selected: YY1 (Yin Yang 1), CREB (c-AMP-response-element-binding protein), and SP1 (Specificity Protein 1). YY1 is a multifunctional transcription factor, ubiquitously expressed^30^, that could cause a looser conformation of chromatin allowing the access to other transcription factors^31^. In addition, it is able to bind CREB, which is activated by phosphorylation in neurons in conditions of hypoxia and oxidative stress by CREB-kinases^32^,^33^. Usually, CREB assemble a transcriptional complex able to advance histone acetylation that alters the conformation of chromatin^33^. SP1 is essential for cell growth and differentiation^34^. In particular in neurons, SP1 DNA-binding complex is induced by oxidative stress, especially as early response to cell stress^35^. We can suppose that in consequence of oxidative stress, YY1 gives an early response binding DNA and inducing looser conformation of chromatin; this helps the connections of CREB and SP1. Then, they connect to transcription machinery resulting in *FTMT* expression. The control of the expression by CREB and SP1 may explain the peculiarity of FtMt pattern of expression detected in mouse that highlighted the presence of protein exclusively in cell type with high energy demand that are more susceptible to ROS formation^20^. From the analysis of DNA region containing the inhibitor elements, we selected: GATA binding protein 2 (GATA2), Forkhead-box protein A1 (FoxA1) and CCAAT-enhancer binding proteins beta (C/EBP β). All of them play essential role in the development and differentiation^36^,^37^,^38^,^39^, thus they might act as inhibitors for *FTMT* during the differentiation process of tissues where its expression is not necessary. In particular, GATA2 is a key transcriptional regulator of haematopoiesis, highly expressed in pluripotent hematopoietic stem cells and in early erythroid cells^40^. In physiological condition FtMt is not detectable in erythroid cells, while it is demonstrated that in pathological condition, like myelodysplastic syndrome with ring sideroblasts (MDS-RARS), it is specifically highly expressed in the ring- syderoblasts^4^,^21^,^28^. Actually, it exists a close correlation between the presence of FtMt and the iron specific Prussian blue staining indicating that mitochondrial ferritin is the form of iron deposited in perinuclear mitochondria of ring sideroblasts^28^.

Interestingly, recent studies have demonstrated that subjects carrying GATA2-sequence variations have high risk to develop MDS^40^, suggesting that this pathogenetic event might be responsible for the up-regulation of FtMt in the disorder, where we have shown to interfere with JAK2/STAT5 pathways leading to ineffective erythropoiesis^27^,^28^.

The ChIP experiments evidenced the existence of a complex in which the different transcriptional factors closely interact, suggesting that they could be simultaneously involved in up/down regulation of the *FTMT* expression. Further experiments are needed to clarify in deeper detail this mechanism.

In addition to the control of *FTMT* expression by transcription factors, we also demonstrated the presence of an epigenetic control that might enhance the tissue-specific *FTMT* gene silencing. Indeed, previous data showed that the level of FtMt is critical for maintaining cellular iron homeostasis^8^,^9^,^27^. When protein is expressed at high level it acts as an iron sink in mitochondria causing cytosolic iron deprivation, for this reason its amount has to be strictly regulated to preserve cellular iron balance. However, it is also demonstrated that its role is important during oxidative damage^10^,^11^. Thus, its physiological amount must be a compromise between its detrimental and beneficial action, and FtMt requirement can be different, based on specificity of type of cells.

To investigate the possibility to modulate the *FTMT* epigenetic control in pathological models, we chose FRDA, a neurological disorder caused by the deficiency of frataxin (FXN), a mitochondrial iron-chaperon involved in iron sulfur cluster biosynthesis^41^. Here, the protective role of FtMt was demonstrated^11^, due to its beneficial effect on iron-dependent chronic oxidative stress that characterize the disease, and where the action of epigenetic therapy was already shown^42^. In fact, in FRDA model, HDAC inhibitor leads to an increased expression of frataxin mRNA^42^ via the inhibition of deacetylation^43^. In a previous work, the presence of FtMt was revealed in FRDA fibroblasts by immunoblotting assay, using an anti-heart ferritin polyclonal antibody with low specificity for FtMt^44^. Differently, we were not able to identify FtMt in our control and FRDA fibroblasts under basal condition, whereas, it was feasible to reveal *FTMT* expression in control and FRDA fibroblasts by the combined treatment with the inhibitor of deacetylation and demethylating agents (Fig. 4). In these conditions, FXN mRNA was not increased in FRDA fibroblasts (not shown), suggesting that the HDAC inhibitor used was not adequate, both for type and concentration. In fact, it was demonstrated that it is necessary a combined inhibition of HDAC 1, 2 and 3 to up-regulate FXN gene^45^. However, the condition to up-regulate *FTMT* is milder than the one necessary to induce FXN, thus, we proposed to take in consideration an epigenetic therapy in FRDA patients to increase at least *FTMT* expression, since it could be useful to help protecting these patients from mitochondrial oxidative stress.

In conclusion, even if further study has to be done to identify the complete transcription machinery responsible of *FTMT* expression and silencing, we identified the promoter region of *FTMT* and we demonstrated a double opposite control of its expression. We located specific regulatory sequences and recognized the respective positive and negative transcription factors. Furthermore, we confirmed that epigenetic factors play a role in the control of *FTMT* expression. We hypothesized that methylated cytosines in the *FTMT* GC islands are recognized by methyl binding protein MeCP2 that can recruit histone de-acetylases, which maintain condensed chromatin. The reagents Aza and NaB make the DNA accessible to transcription factors and activate *FTMT* expression. Our findings sustain that the moderate induction of *FTMT* expression by epigenetic therapy could be useful in disease characterized by oxidative stress, as FRDA.

## Materials and Methods

### Database research

In order to understand the methylation status of *FTMT* promoter region, we exploited the data available from the ENCODE project^46^. In particular, we checked for methylation status of CpG islands using the track “CpG Methylation by Methyl 450 K Bead Arrays from ENCODE/HAIB” and extracted the genomic sequence and positions of probes mapping up to 3 kb upstream of *FTMT* promoter.

The sequence comparisons were performed by ECR Browser^47^ and ClustalW2^48^. The transcription binding sites were analyzed by MatInspector (Genomatix Software GmbH)^49^ and by PROMO (Alggen)^50^.

### Plasmid construction

The pcDNA3-FtMt plasmid, encoding the entire FtMt protein precursor, has been described previously^8^. Plasmid containing the promoter region and fragments were amplified from human genomic DNA by PCR using primers (Table 1) containing XhoI and HindIII recognition sites. The promoter fragments (−1884 bp/−1 bp) were cloned into a promoterless luciferase reporter vector, pGL2. We designed oligonucleotides to amplify fragments deleted at 5′ (−902/+9, −491/+9; −217/+9, −91/+9) or at 3′ (−1884/−217, −1884/−464,−1884/−521, −1884/−631, −1884/−777, −1884/−874, −1884/−1128) (Table 1).

### Cell culture, transfection and luciferase assay

HeLa cells (ATCC, cat. n° CCL-2) and a fibroblast cell line (ATCC, cat. n° PCS-201-012) for control and derived from one FRDA patient (1037SS previously described in^11^) were grown in high glucose DMEM supplemented with 10% FBS, penicillin/streptomycin antibiotics and glutamine at 37° and 5% CO_2_. The human erythroleukemic K562, (ATCC, cat. n° CCL-243) cells were cultured in RPMI supplemented with 10% FBS, penicillin/streptomycin antibiotics and glutamine at 37° and 5% CO_2_. HeLa and K562 cells were transiently transfected using Lipofectamine 2000 (Invitrogen, San Giuliano Milanese, Italy). HeLa cells were chosen because they are an easier transfection cell system, while K562 cells because they have been previously described as a model of low *FTMT* expression in basal condition^19^. Epigenetic evaluation was done by treatment of cells for 72 h with 5 μM of 5-Aza-2′-deoxycytidine (decitabine or Aza, Sigma-Aldrich, Milan, Italy), a demethylating agent, and with 1 mM of Sodium Butyrate (NaB, Sigma-Aldrich, Milan, Italy), an histone deacetylase inhibitor (HDAC inhibitor).

Luciferase assay was performed following 48 h transfection. Renilla luciferase and firefly luciferase activities were analyzed using the Dual-Luciferase® Reporter Assay System (Promega, Milan, Italy) following the manifacturer’s instructions. The firefly luciferase activities were normalized using the Renilla luciferase activity and luciferase vector pGL2-basic values were used as negative controls.

### Chromatin Immuno Precipitation (ChIP)

Formaldehyde crosslinking and chromatin immunoprecipitation assay on K562 cells, equivalent to 3 × 10^7^ for each ChIP was performed as previously described^51^. Chromatin samples were immunoprecipitated O/N at 4 °C rotating by incubation with the following antibodies: rabbit polyclonal to CREB (ab31387), rabbit polyclonal to SP1 (ab13370), rabbit polyclonal to YY1 (ab38422), rabbit polyclonal to FOXA1 (ab23738), rabbit polyclonal to GATA2 (ab22849), rabbit monoclonal to CEBP Beta (ab32358, Abcam, Cambridge, UK) and rabbit IgG (Cell Signaling Technology, Leiden, The Netherlands). Protein G-Dynabeads were washed in PBS, 0.5% BSA O/N at 4 °C. The day after, IP samples were added to Dynabeads and incubated for 2 h at 4 °C rotating. The beads were then washed 3 times for 10 min each at 4 °C rotating. After the last wash, the supernatant was removed, 120 μl of decrosslink solution (100 mM NaHCO_3_, 1% SDS) was added in each sample and incubated O/N at 65 °C with shaking. The day after, the samples were incubated with Proteinase K (0.4 mg/ml) for 2 h at 55 °C with shaking and subsequently with RNAse (0.15 mg/ml) for 2 h at 37 °C with shaking. DNA samples were purified by GE Healthcare PCR purification kit, following the kit instructions (GE Healthcare, Milan, Italy). The DNA was eluted in 50 μl of dd H_2_O and 1–2 μl were used for real-time polymerase chain reaction (qRT-PCR). qRT-PCR was performed by SYBR green TaqMan® with primers designed to specifically amplify human *FTMT* promoter and more specifically activator and inhibitor regions (Table 2) using ABI7900HT fast real time PCR system (Applied Biosystems, Monza, Italy).

### Methylation specific PCR

DNA methylation was analyzed using bisulfite-modified genomic DNA. Aliquots of 500 ng of isolated genomic DNA were bisulfite modified using the EZ Methylation Bisulfite Kit (Zymo Research, Pero, Italy) according to manufacturer’s instructions. The applied primer pairs (Table 2) gave PCR products size of 187 bp for methylation sequences and of 186 bp for un-methylated sequences. Primers were applied to a final concentration of 0.5 μM. Cycling conditions were as follows: 10 min at 95 °C, followed by 34 cycles of 30 s at 94 °C, 30 s at 55 °C (methylated sequences)/57 °C (un-methylated sequences), and 45 s at 72 °C, and a final 7 min extension step at 72 °C. After amplification, PCR products were subjected to 2% agarose gel electrophoresis to control and establish the purity of amplicones. The PCR products were purified using QIAquick Gel Extraction Kit (QIAGEN, Milan, Italy) according to the manufacturer’s instructions. Five ng of PCR products were then prepared for sequencing in order to verify amplicones specificity. Sequences were obtained using ABI PRISM® 3130XL automatic sequencer (Applied Biosystems).

### RNA extraction and qRT-PCR

Total RNA was extracted from cells using the RNeasy Mini Kit (QIAGEN, Milan, Italy) as per the manufacturer’s directions. RNA quantity was assessed with Nanodrop (NanoDrop Technologies, Wilmington, DE) and equal amounts of total RNA (4 μg) was incubated with 1 U DNaseI (Invitrogen, Monza, Italy); it was reverse transcribed using the Superscript III RT kit (Invitrogen, Monza, Italy). Samples without reverse transcriptase were included as negative controls. The resulting cDNA was preamplified by TaqMan PreAmp Master Mix Kit (Applied Biosystems) as per the manufacturer’s directions. qRT-PCR was performed by TaqMan® Gene Expression Assay using ABI7900HT fast real time PCR system. *FTMT* mRNA expression is relative to housekeeping gene *HPRT1* (Hypoxanthine Phosphoribosyltransferase1). (FTMT: Hs00893202_s1; HPRT1: Hs02800695, both Applied Biosystems). To verify Aza treatment efficacy, primers were designed to specifically amplify human *INSL6* and *GAPDH* as housekeeping (Table 2) and qRT-PCR was performed by Sybr green TaqMan® with, using ABI7900HT fast real time PCR system.

### Statistical analyses

The data, except where otherwise indicated, are reported as the mean +/− SD values or as representative of at least three independent experiments with similar results. Data were analyzed using GraphPad Prism. In general, for normally distributed data two tailed unpaired one- or two-way ANOVA followed by Bonferroni post test was used. For non-normally distributed data, Mann-Whitney rank sum test was used. *^,^ ** and *** indicate p < 0.05, p < 0.01 and p < 0.001, respectively. A p value < 0.05 was considered statistically significant.

## Additional Information

**How to cite this article**: Guaraldo, M. *et al*. Characterization of human mitochondrial ferritin promoter: identification of transcription factors and evidences of epigenetic control. *Sci. Rep.* **6**, 33432; doi: 10.1038/srep33432 (2016).

## Figures and Tables

**Table 1 t1:** Primers for plasmids construction.

**Plasmid**	**Forward Oligonucleotide (5′ to 3′)**	**Reverse Oligonucleotide (5′ to 3′)**
pGL2Mt (1893 bp: −1884 +9)	MITA: ACGTAGCCTCGAGATGTGGTTGACAGAAGGCAGGTGCAAACA	MITC: CGATACAAGCTTAGCGCCGCCTCCTTGGAAGT
pGL2MtA (226 bp: −217 +9)	MIT1: ACGTAGCCTCGAGTGCGCTGGCCTCCGCCTAGA	MITC
pGL2MtC (100 bp: −91 +9)	MIT2: ACGTAGCCTCGAGTGCCTAGGGCCACGTTCTGATCAG	MITC
pGL2MtF (911 bp: −902 +9)	MIT5: ACGTAGCCTCGAGACGGAGTCTTGCTCTGTCGCCCA	MITC
pGL2MtG (500 bp: −491 +9)	MIT6: ACGTAGCCTCGAGATGTACACTTGCTTTGGATGTGGACCT	MITC
pGL2MtH (1667 bp: −1884 −217)	MITA	MITH1: CTATACAAGCTTGATGCCCAGTCACGATTCCGT
pGL2MtM (1010 bp: −1884 −874)	MITA	MTM: CTATACAAGCTTCAGCCTGGGCGACAGAGCAAGA
pGL2MtM1 (1107 bp: −1884 −777)	MITA	MTM1: CTATACAAGCTTTGGTGGCAGGCTGCTGTAGT
pGL2MtM2 (1253 bp: −1884 −631)	MITA	MTM2: CTATACAAGCTTATACTGTAGGCCGGGTGCCGT
pGL2MtM3 (1363 bp: −1884 −521)	MITA	MTM3: CTATACAAGCTTTGGAAACAATTGCCAGCATAGTGGGA
pGL2MtN (1420 bp: −1884 −464)	MITA	MITN: CTATACAAGCTTAGGTCCACATCCAAAGCAAGTGTACATA
pGL2MtO (756 bp: −1884 −1128)	MITA	MITO: CTATACAAGCTTAAGGGTAAGCACGCAGAAGAAAGTTCA

**Table 2:** Primers for quantitative real time PCR.

**Plasmid**	**Forward Oligonucleotide (5**′ **to 3**′)	**Reverse Oligonucleotide (5**′ **to 3**′)
*FTMT*	RT1: TATGCGTCCTACGTGTACT	RT2: TGTTCCGGCTTCTTGATGT
*GAPDH*	GAPDHf: TCCCATCACCATCTTCCAG	GAPDHr: ATGAGTCCTTCCACGATACC
*INSL6*	INSL6f: AAAAACTCTGCGGCCATGC	INSL6r: CCCAACTGTTTACTGCTTCTTCC
*FTMT* activator	FTMTf: GAGTGCAGTGGCTCGATCT	FTMTr: GTGGCAGGCTGCTGTAGT
*FTMT* inhibitor	FWI2: CTAACCTTGTGATCCGCC	RVI5: ATGAGATACTGTAGGCCGG
*FTMT* total regulatory	FTMTf: GAGTGCAGTGGCTCGATCT	RVI5: ATGAGATACTGTAGGCCGG
Methylated sequences	TCGATTTTACGTTAAGAGGGTC	GATCTACCTAAAATCCAAAAAACG
Un-methylated sequences	TGATTTTATGTTAAGAGGGTTGG	AATCTACCTAAAATCCAAAAAACACC
